# Traditional Chinese medicine in sepsis-associated PICS: potential immunometabolic mechanisms and translational perspectives

**DOI:** 10.3389/fimmu.2026.1827446

**Published:** 2026-08-17

**Authors:** Pochen Li, Fangyu Yu, Er Hong, Yan Wu, Danxia Ge, Qianping Zhang, Yujiao Li, Yang Wu

**Affiliations:** 1 Intensive Care Unit, Ningbo Municipal Hospital of Traditional Chinese Medicine (TCM), Affiliated Hospital of Zhejiang Chinese Medical University, Ningbo, China; 2 Department of Respiratory, Ningbo Municipal Hospital of Traditional Chinese Medicine (TCM), Affiliated Hospital of Zhejiang Chinese Medical University, Ningbo, China

**Keywords:** immune dysregulation, immunometabolism, persistent inflammation-immunosuppression-catabolism syndrome (PICS), sepsis, traditional Chinese medicine, Xuebijing

## Abstract

Persistent inflammation, immunosuppression, and catabolism syndrome (PICS) has emerged as an important contributor to long-term morbidity among survivors of sepsis. This syndrome is characterized by sustained immune dysregulation, metabolic disturbances, and progressive skeletal muscle wasting, reflecting a failure of coordinated recovery following severe infection. Although advances in critical care have improved early survival, effective strategies targeting the complex immunometabolic alterations underlying PICS remain limited. Traditional Chinese medicine (TCM) has increasingly been investigated as an adjunctive approach in critical care. Randomized clinical studies have reported potential benefits of several Chinese herbal injections in patients with sepsis, particularly Xuebijing injection, which has been associated with improvements in short-term clinical outcomes and inflammatory markers. Experimental studies further suggest that bioactive compounds contained in herbal formulations may influence multiple biological processes relevant to immune regulation and metabolic homeostasis. However, most available evidence has been derived from studies conducted during the acute phase of sepsis rather than the prolonged recovery phase in which PICS develops. In addition, the absence of a standardized diagnostic definition for PICS, together with variability in biomarker thresholds, diagnostic timing, and outcome measures across studies, limits comparability and may introduce heterogeneity in patient selection. These factors constrain the interpretation and generalizability of current findings. Accordingly, the potential role of TCM in PICS should be interpreted with caution. Rather than providing definitive therapeutic recommendations, this review critically synthesizes the currently available evidence, identifies important knowledge gaps, and proposes a conceptual framework to guide future mechanistic and translational studies in post-sepsis recovery.

## 1 Introduction

Advances in critical care medicine have substantially improved short-term survival among patients with severe sepsis and septic shock (1–3). However, survival from the acute episode is frequently followed by a prolonged trajectory of morbidity rather than full physiological recovery. A considerable proportion of sepsis survivors develop persistent immune and metabolic disturbances that predispose them to recurrent infections, functional decline, and increased long-term mortality.

Persistent inflammation, immunosuppression, and catabolism syndrome (PICS) has been proposed as a conceptual framework to describe these chronic complications following severe infection (4–6). Patients with PICS typically exhibit sustained low-grade inflammation, impaired innate and adaptive immune responses, and progressive protein-energy catabolism, often manifesting clinically as chronic critical illness, prolonged hospitalization, skeletal muscle wasting, and reduced quality of life. However, the definition and diagnostic criteria of PICS remain incompletely standardized across studies (7).

Current management strategies for sepsis primarily target the acute phase of illness, including antimicrobial therapy, hemodynamic stabilization, and organ support (8, 9). Although these interventions reduce early mortality, they have limited impact on the long-term immunological and metabolic disturbances that characterize PICS. As a result, there is increasing interest in therapeutic approaches capable of modulating multiple biological pathways involved in post-sepsis recovery.

Traditional Chinese medicine (TCM) has attracted increasing attention as a potential adjunctive approach in the management of sepsis. Several Chinese herbal injections have been evaluated in randomized clinical studies, with some evidence suggesting improvements in short-term clinical outcomes and inflammatory markers, particularly for Xuebijing injection. Experimental studies further indicate that bioactive compounds within herbal formulations may influence pathways related to immune regulation, mitochondrial function, endothelial integrity, and metabolic homeostasis.

However, it is important to note that most available studies have been conducted during the acute phase of sepsis rather than during the prolonged recovery phase in which PICS develops. In addition, methodological heterogeneity, variability in diagnostic definitions, and challenges related to standardization and quality control limit the interpretation and generalizability of current evidence.

Given the scarcity of direct clinical evidence in patients with established PICS, the purpose of this Mini Review is not to establish therapeutic efficacy of traditional Chinese medicine but rather to critically synthesize the currently available clinical, experimental, and mechanistic evidence, identify important knowledge gaps, and provide a conceptual immunometabolic framework to guide future translational research. Particular emphasis is placed on distinguishing direct evidence from indirect evidence, critically evaluating the strengths and limitations of the current literature, and outlining priorities for future investigation.

## 2 Literature search strategy

A structured but non-systematic literature search was conducted to identify relevant studies on sepsis, PICS, and traditional Chinese medicine. Electronic databases, including PubMed, Embase, Web of Science, CNKI, and Wanfang, were searched from database inception to January 2026.

Search terms included combinations of keywords such as “sepsis”, “persistent inflammation, immunosuppression, and catabolism syndrome” or “PICS”, “chronic critical illness”, “immunometabolism”, “traditional Chinese medicine”, “herbal medicine”, and “Xuebijing”.

Relevant randomized controlled trials, observational studies, and experimental research articles were considered. Additional references were identified through manual screening of the reference lists of included studies.

Given the narrative nature of this Mini Review, studies were selected according to their scientific relevance, methodological quality, and contribution to understanding either direct clinical evidence in PICS, indirect clinical evidence from acute sepsis, or mechanistic insights potentially relevant to post-sepsis immunometabolic recovery.

## 3 Clinical evidence of TCM in sepsis and implications for PICS

### 3.1 Direct clinical evidence in PICS

For clarity, the available literature is discussed according to its level of relevance to PICS, including direct clinical evidence in established PICS, indirect clinical evidence from acute sepsis, and mechanistic or preclinical evidence. This hierarchical approach was adopted to avoid overinterpretation of findings obtained outside the PICS setting.

Direct clinical evidence evaluating the effects of traditional Chinese medicine (TCM) specifically in patients with established PICS remains limited. Available studies are predominantly derived from single-center observational analyses and small-scale interventional investigations, many of which have been reported in the Chinese-language literature.

Observational studies have focused on characterizing the clinical features, risk factors, and syndrome patterns of PICS in patients with sepsis or severe pneumonia (10, 11). These studies consistently show that patients who develop PICS exhibit more pronounced inflammatory responses, impaired immune status, including reduced lymphocyte counts and altered monocyte-related indices, and poorer nutritional profiles than non-PICS patients. In addition, PICS is associated with prolonged ICU stay, greater healthcare burden, and significantly higher long-term mortality. Identified risk factors include higher disease severity scores, such as APACHE II and SOFA, elevated inflammatory markers such as C-reactive protein, and indicators of metabolic dysfunction. Collectively, these findings support the clinical relevance of PICS as a distinct post-sepsis phenotype and provide a basis for further investigation.

From an integrative medicine perspective, several studies have explored the evolution of traditional Chinese medicine syndrome patterns in PICS. These analyses suggest that PICS is commonly characterized by a pattern of “deficiency mixed with excess,” with a dynamic transition toward predominance of deficiency syndromes, such as qi deficiency and yang deficiency, over time (12–14). Such observations are broadly consistent with the progression from acute inflammatory responses to chronic immune suppression and catabolic dysfunction described in modern medicine (15).

Interventional evidence in PICS remains scarce. A prospective study evaluating a classical herbal formulation, Sijunzi decoction, in patients with PICS reported improvements in gastrointestinal function and modulation of immune cell subsets, including increases in CD3+, CD4+, and CD8+ T-lymphocyte levels (16). These findings suggest that TCM interventions may influence immune regulation and functional recovery during the PICS phase. However, the study was limited by its small sample size, short follow-up duration, and lack of standardized endpoints.

Overall, direct evidence in PICS remains preliminary and heterogeneous. Although insufficient to support definitive conclusions, it provides initial clinical observations that warrant further investigation.

### 3.2 Clinical evidence in acute sepsis

In contrast to the limited evidence available in PICS, a larger body of clinical research has evaluated traditional Chinese medicine interventions during the acute phase of sepsis. Among these, Chinese herbal injections, particularly Xuebijing injection, have been the most extensively studied (17–20).

The EXIT-SEP trial represents the most robust clinical evidence in this field. This multicenter, randomized, double-blind, placebo-controlled trial enrolled 1,817 patients with sepsis admitted to intensive care units in China. Treatment with Xuebijing was associated with a significant reduction in 28-day all-cause mortality compared with placebo (17). In addition, improvements were observed in several secondary outcomes, including ICU mortality, in-hospital mortality, ICU-free days, and ventilator-free days within 28 days.

In terms of methodological quality, the EXIT-SEP trial demonstrates a relatively low risk of bias. The study employed a rigorous randomized, double-blind, placebo-controlled design, together with appropriate sample-size calculation and intention-to-treat analysis, thereby strengthening the internal validity of the findings.

However, several factors should be considered when interpreting these results. First, the study population consisted exclusively of patients from China, which may limit external generalizability across different healthcare systems and ethnic groups. Second, although standard-of-care treatment was provided, variations in clinical practice across participating centers may have introduced heterogeneity. Third, as with many trials involving complex herbal formulations, complete blinding of treatment effects may be challenging because of potential differences in administration or clinical perception.

In addition, publication bias cannot be entirely excluded, particularly in the context of TCM-related clinical research, where positive findings may be preferentially reported. Taken together, although the EXIT-SEP trial provides relatively robust evidence for short-term efficacy, these limitations should be considered when extrapolating its findings to broader populations and to long-term outcomes such as PICS.

Other clinical studies of TCM interventions in sepsis have reported improvements in inflammatory markers and organ function parameters. However, these studies are heterogeneous in design, sample size, and methodological rigor. Issues such as inadequate blinding, variability in comparator treatments, and differences in standard-of-care practices further complicate interpretation.

Importantly, these clinical trials were conducted during the acute phase of sepsis and did not directly evaluate outcomes related to the prolonged recovery phase in which PICS develops. Therefore, although these findings suggest that TCM interventions may modulate inflammatory and immune responses in early sepsis, their applicability to PICS remains uncertain.

### 3.3 Mechanistic and preclinical evidence

Preclinical and mechanistic studies provide additional insights into the potential biological effects of TCM interventions. Experimental data suggest that certain bioactive compounds may regulate inflammatory signaling pathways, modulate immune responses, and influence mitochondrial function and metabolic homeostasis. These mechanisms are broadly relevant to the immunometabolic disturbances observed in sepsis.

In addition, theoretical and observational studies within the TCM framework have proposed that PICS corresponds to syndromes characterized by deficiency of vital energy, impaired organ function, and accumulation of pathological factors such as phlegm and blood stasis (10, 11, 14). These concepts emphasize the interaction between immune dysfunction, metabolic imbalance, and impaired recovery.

However, most mechanistic evidence is derived from experimental models or indirect clinical observations rather than from studies conducted specifically in patients with PICS. Accordingly, such evidence should be regarded as indirect and hypothesis-generating rather than confirmatory.

### 3.4 Implications for PICS

Taken together, current evidence suggests that TCM interventions may influence biological processes relevant to inflammation, immune regulation, and metabolic homeostasis. These mechanisms are conceptually aligned with key features of PICS, including persistent inflammation, immune suppression, and catabolic dysregulation.

From an integrative perspective, these findings support the hypothesis that TCM may act on interconnected immunometabolic pathways rather than isolated targets, which is consistent with the complex and multifactorial nature of PICS. In particular, the potential modulation of inflammatory signaling, mitochondrial function, and immune cell activity provides a theoretical framework through which TCM might influence post-sepsis recovery trajectories (21).

However, it is important to emphasize that most available evidence is derived from studies conducted during the acute phase of sepsis or from preclinical models (22). Direct clinical evidence in patients with established PICS remains limited, and existing studies are characterized by heterogeneity in diagnostic criteria, study design, and outcome measures, which may contribute to variability in reported findings.

Overall, the currently available evidence suggests that TCM may influence multiple immunometabolic pathways relevant to post-sepsis recovery. However, most supporting evidence originates from acute-phase sepsis studies or preclinical investigations rather than patients with established PICS. Consequently, the available literature should be interpreted cautiously.

At present, TCM cannot be considered an evidence-based therapy for PICS; instead, current findings provide preliminary clinical signals together with mechanistic plausibility that should be regarded as hypothesis-generating rather than evidence-confirming.

Importantly, this evidence hierarchy highlights a critical translational gap between mechanistic plausibility and clinically validated therapeutic efficacy in patients with established PICS. To facilitate interpretation of the current evidence base, the principal studies are critically summarized in **Table 1** according to evidence category, methodological characteristics, strengths, limitations, and their potential relevance to PICS.

**Table 1:** Critical appraisal of the current evidence supporting traditional Chinese medicine in sepsis-associated PICS.

Evidence category	Representative studies	Level of evidence	Major strengths	Major limitations	Clinical relevance to PICS
Direct clinical evidence in established PICS	Niu et al. (2014) (16); Liu et al. (2021) (12); Cao (2024) (11); Yuan (2025) (10)	Preliminary clinical evidence	Direct evaluation of patients with established PICS; provides initial observations regarding immune function, nutritional status, gastrointestinal recovery, and TCM syndrome evolution	Small sample sizes; predominantly single-center studies; heterogeneous diagnostic criteria; short follow-up; lack of standardized endpoints	Suggests potential benefit but remains insufficient to establish therapeutic efficacy
Clinical evidence in acute sepsis	EXIT-SEP trial (2023) (17); Li et al. (2018) (19); Shi et al. (2017) (20); Li et al. (2021) (18)	Indirect clinical evidence	Includes multicenter randomized controlled trials and meta-analyses demonstrating improved short-term outcomes and modulation of inflammatory responses	Conducted during acute sepsis rather than established PICS; limited long-term follow-up; uncertain external validity for post-sepsis recovery	Supports biological plausibility but should not be directly extrapolated to patients with established PICS
Mechanistic and preclinical evidence	Hydroxysafflor yellow A; Paeoniflorin; Tanshinone IIA; Ferulic acid	Preclinical evidence	Provides mechanistic rationale through regulation of NF-κB, HIF-1α, AMPK, mTOR, mitochondrial function, oxidative stress, and immune metabolism	Predominantly derived from experimental models; limited clinical validation; uncertain translational relevance	Supports mechanistic plausibility rather than clinically validated efficacy
Overall interpretation	Integrated assessment of current literature	Hypothesis-generating evidence	Consistent biological rationale across mechanistic studies with supportive indirect clinical observations	Direct evidence specific to PICS remains scarce and heterogeneous	A critical translational gap persists between mechanistic plausibility and clinically validated therapeutic efficacy, highlighting priorities for future prospective studies

Critical appraisal of the current evidence supporting traditional Chinese medicine (TCM) in sepsis-associated persistent inflammation, immunosuppression, and catabolism syndrome (PICS). The available evidence is stratified according to its relationship to PICS, including direct clinical evidence, indirect clinical evidence from acute sepsis, and mechanistic or preclinical investigations. This evidence hierarchy highlights the strengths, limitations, level of evidence, and clinical relevance of the current literature while emphasizing the translational gap between mechanistic plausibility and clinically validated therapeutic efficacy.

## 4 Mechanistic insights relevant to PICS

The pathophysiology of PICS is increasingly recognized as a consequence of disrupted immunometabolic homeostasis following sepsis, in which persistent inflammation, immunosuppression, and catabolic metabolism are mechanistically interconnected rather than independent processes. This integrated framework suggests that immune dysfunction in PICS is fundamentally shaped by alterations in cellular metabolic programs.

Persistent low-grade inflammation in PICS is partly sustained by ongoing activation of pattern-recognition receptor-mediated signaling pathways. Even after apparent resolution of infection, damage-associated molecular patterns (DAMPs) released from injured tissues can continue to activate nuclear factor-κB (NF-κB), leading to prolonged production of pro-inflammatory cytokines such as IL-6 and TNF-α. Importantly, NF-κB activation is closely coupled with metabolic reprogramming, particularly a shift toward aerobic glycolysis in activated immune cells, indicating that inflammatory signaling is metabolically sustained rather than transient (23, 24).

Hypoxia-inducible factor-1α (HIF-1α) serves as a key mediator linking metabolic reprogramming to inflammatory responses. Under conditions of cellular stress, HIF-1α enhances glycolytic flux and promotes transcription of pro-inflammatory genes, thereby reinforcing a pro-inflammatory phenotype. In parallel, mechanistic target of rapamycin (mTOR) integrates nutrient sensing with immune activation and supports anabolic metabolism in activated immune cells. Together, these pathways contribute to the maintenance of a metabolically driven inflammatory state in PICS.

In contrast, AMP-activated protein kinase (AMPK) functions as a critical metabolic checkpoint that counterbalances excessive inflammation. Activation of AMPK promotes oxidative phosphorylation, enhances mitochondrial biogenesis, and suppresses NF-κB-mediated inflammatory signaling. Dysregulation of AMPK signaling during sepsis recovery may impair the transition from inflammatory to restorative metabolic states, thereby contributing to persistent immunometabolic imbalance.

Immunosuppression represents another defining feature of PICS and is characterized by lymphocyte apoptosis, impaired antigen presentation, and expansion of myeloid-derived suppressor cells (MDSCs) (25, 26). Reduced expression of monocytic human leukocyte antigen-DR (HLA-DR) reflects impaired antigen-presenting capacity and is associated with increased susceptibility to secondary infection. In addition, activation of immune checkpoint pathways contributes to T-cell exhaustion and long-term immune dysfunction. These findings indicate that immune suppression in PICS is actively regulated rather than merely a passive consequence of inflammation.

Recent studies have demonstrated that innate immune cells undergo long-term functional reprogramming following sepsis, a phenomenon referred to as trained immunity. This process is mediated by coordinated metabolic and epigenetic changes, including alterations in glycolysis, tricarboxylic acid cycle intermediates, and histone modifications. Importantly, trained immunity can result in divergent outcomes. Whereas adaptive “training” may enhance antimicrobial responses, maladaptive reprogramming may lead to immune tolerance, characterized by reduced cytokine production and impaired responsiveness. The balance between these states appears to depend on the metabolic context and inflammatory burden.

Mitochondrial dysfunction represents a central link between immunometabolic disturbance and organ-level consequences in PICS. Impaired mitochondrial biogenesis, reduced ATP production, and increased oxidative stress contribute to immune cell dysfunction and skeletal muscle catabolism (27). In addition, mitochondrial damage-associated molecular patterns may further amplify inflammatory signaling, creating a self-sustaining cycle of immune dysregulation. These observations highlight the critical role of mitochondrial integrity in coordinating immune and metabolic recovery.

Endothelial injury and disruption of the gut–organ axis further exacerbate immunometabolic imbalance. Glycocalyx degradation impairs microcirculatory perfusion and promotes leukocyte adhesion, while intestinal barrier dysfunction facilitates microbial translocation and sustained immune activation (28, 29). Collectively, these interconnected pathways form an integrated immunometabolic network that underlies the persistence of inflammation, immune dysfunction, and catabolism in PICS.

## 5 Potential mechanistic basis of TCM in PICS

Compared with the relatively well-characterized immunometabolic mechanisms of PICS, the molecular basis of traditional Chinese medicine remains less precisely defined. Nevertheless, accumulating pharmacological evidence has begun to identify specific bioactive constituents within commonly used herbal formulations that may interact with key pathways involved in immune and metabolic regulation (30–32).

Xuebijing injection, one of the most extensively studied traditional Chinese medicine interventions in sepsis, contains multiple active components, including hydroxysafflor yellow A, paeoniflorin, ferulic acid, and tanshinone IIA (17, 18). Experimental studies suggest that these compounds exert anti-inflammatory effects, at least in part, through inhibition of NF-κB signaling and reduction of pro-inflammatory cytokine production. For example, hydroxysafflor yellow A has been reported to attenuate inflammatory responses and improve microcirculatory function, whereas paeoniflorin may modulate immune responses by influencing macrophage activation and cytokine balance (33, 34). These observations are consistent with pathways implicated in sepsis-associated immune dysregulation.

At the level of immunometabolism, several bioactive compounds derived from TCM have been associated with regulation of mitochondrial function and cellular energy metabolism. Tanshinone IIA, a lipophilic diterpene quinone, has been reported to improve mitochondrial function and attenuate oxidative stress, with some studies suggesting involvement of AMP-activated protein kinase (AMPK)-related pathways (35, 36). These effects have primarily been observed in experimental models of inflammation and cellular injury.

Similarly, ferulic acid, a phenolic compound widely present in herbal formulations, exhibits well-documented antioxidant properties and has been reported to participate in the regulation of redox balance under inflammatory conditions (37). In addition, pharmacokinetic studies indicate that ferulic acid undergoes rapid absorption and metabolism, which may influence its biological activity *in vivo* (38).

Taken together, these findings suggest that certain herbal constituents may influence metabolic processes relevant to immune cell function. However, it should be noted that most available evidence is derived from preclinical studies, and direct evidence linking these effects to immunometabolic regulation in patients remains limited.

In addition to effects on inflammatory signaling and metabolism, preliminary evidence suggests that herbal interventions may interact with immune regulatory pathways involved in sepsis. Experimental studies have indicated potential modulation of macrophage activation and cytokine responses. However, evidence directly demonstrating restoration of clinically relevant immune markers, such as monocytic human leukocyte antigen-DR (HLA-DR) expression, remains insufficient, particularly in the context of established PICS.

From a pharmacological perspective, the multi-component nature of herbal formulations introduces substantial complexity in pharmacokinetics and pharmacodynamics. Individual compounds differ in bioavailability, metabolic pathways, and tissue distribution. For instance, hydrophilic constituents such as hydroxysafflor yellow A and paeoniflorin exhibit relatively rapid systemic clearance, whereas lipophilic compounds such as tanshinone IIA display distinct absorption and distribution characteristics (39). These pharmacokinetic differences may influence overall biological effects and complicate mechanistic interpretation.

**Figure 1:**
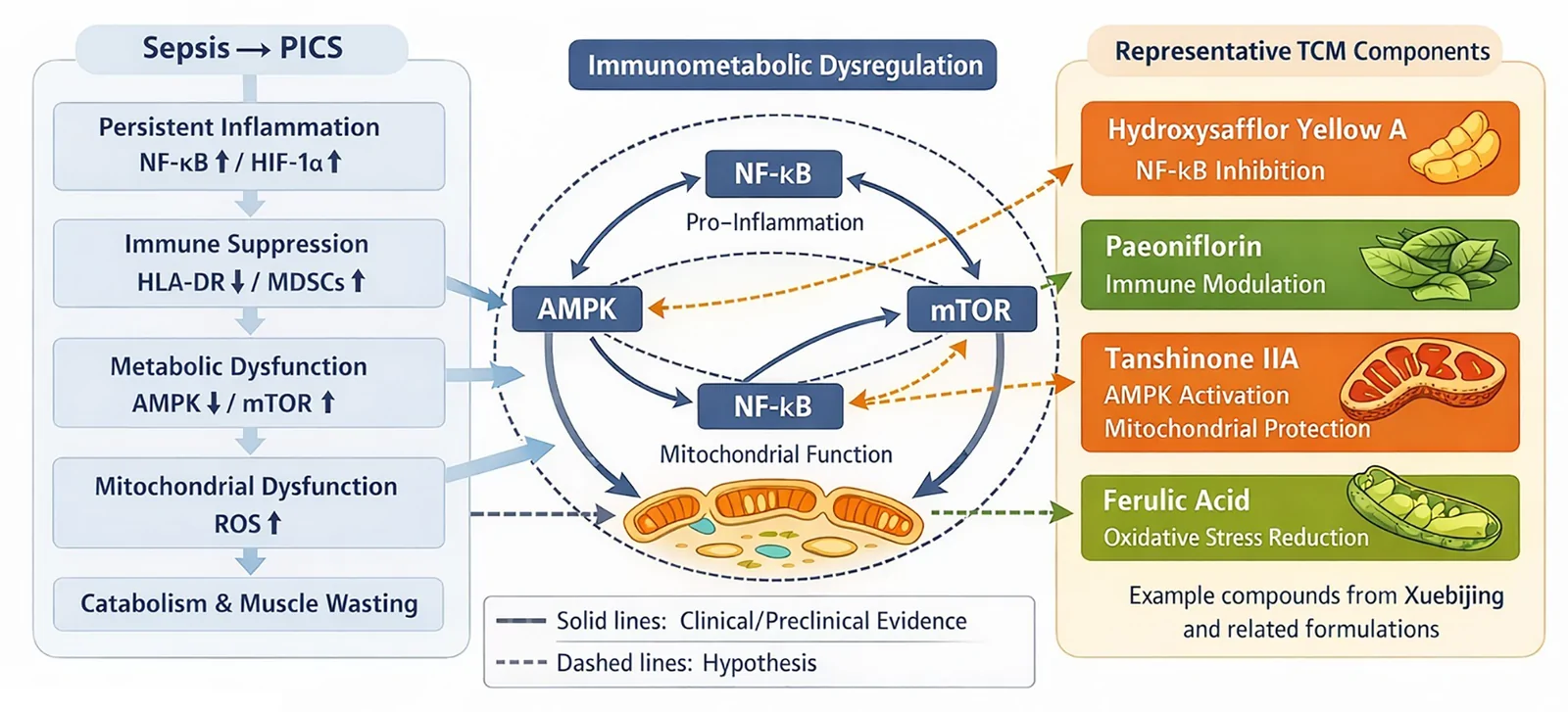
Proposed immunometabolic framework of PICS and potential targets of representative traditional Chinese medicine (TCM) components. PICS is characterized by interconnected dysregulation of inflammatory signaling, immune function, and cellular metabolism, involving pathways such as NF-κB, HIF-1α, AMPK, mTOR, and mitochondrial function. Representative TCM-derived compounds—including hydroxysafflor yellow A, paeoniflorin, tanshinone IIA, and ferulic acid—have been reported to interact with these pathways. However, most supporting evidence derives from preclinical or acute-phase sepsis studies, and relevance to established PICS remains uncertain. Solid lines indicate interactions supported by clinical or preclinical evidence, whereas dashed lines represent hypothesized relationships. Accordingly, the proposed framework should be interpreted as a conceptual model integrating current evidence rather than as confirmation of therapeutic efficacy in established PICS.

Importantly, the current evidence base remains limited by the predominance of preclinical studies and clinical investigations conducted during the acute phase of sepsis. Direct evidence linking specific TCM interventions to immunometabolic regulation in PICS is currently lacking. Therefore, the proposed mechanisms should be interpreted with caution and regarded as hypothesis-generating rather than definitive (**Figure 1**).

## 6 Challenges and limitations

Despite growing interest in traditional Chinese medicine (TCM) as an adjunctive therapy in sepsis, several important limitations should be considered before extending its application to PICS.

Most clinical studies of herbal interventions have focused on short-term outcomes during the acute phase of sepsis, such as 28-day mortality and inflammatory biomarkers (40–43). In contrast, PICS develops over a prolonged period characterized by persistent immune dysfunction, metabolic disturbances, and progressive muscle wasting. As a result, current evidence provides limited insight into whether these interventions influence long-term recovery trajectories (44, 45).

An additional source of uncertainty relates to the lack of a standardized diagnostic definition for PICS. As discussed above, existing studies apply heterogeneous criteria with respect to biomarker thresholds, diagnostic time windows, and combinations of clinical indicators. This variability may lead to inconsistencies in patient selection and contribute to heterogeneity across study populations, thereby introducing potential bias in the interpretation of therapeutic effects (6, 46).

Substantial heterogeneity also exists among sepsis survivors. Immune and metabolic trajectories vary according to factors such as age, comorbidities, infection severity, and treatment exposures. Without biomarker-based stratification or dynamic immune monitoring, clinical trials may fail to identify patient subgroups most likely to benefit from targeted interventions (47, 48).

Safety considerations warrant particular attention, especially in critically ill or post-sepsis populations receiving multiple concomitant therapies. Patients in the ICU are frequently exposed to complex treatment regimens, including antibiotics, vasoactive agents, sedatives, and organ support therapies, which may increase the risk of herb–drug interactions. Although available clinical studies generally report acceptable short-term safety profiles for certain herbal injections, the potential for pharmacokinetic interactions, such as modulation of drug metabolism pathways, and cumulative toxicity remains insufficiently characterized (49).

Quality control and standardization represent additional challenges. Variability in plant sources, extraction processes, and manufacturing conditions may result in differences in chemical composition between batches, potentially affecting both efficacy and safety. Although standardized preparations such as Chinese herbal injections have improved consistency compared with traditional decoctions, concerns regarding batch-to-batch variability and reproducibility remain relevant.

From a translational perspective, regulatory considerations also require careful attention. Outside China, differences in regulatory frameworks, quality control requirements, and approval pathways may limit the broader clinical adoption of herbal formulations. Furthermore, the lack of large-scale, high-quality clinical trials conducted under internationally standardized conditions continues to constrain the generalizability of existing evidence.

More broadly, given that sepsis remains a major global health priority, future translational research should place greater emphasis on long-term recovery outcomes, methodological rigor, and clinically meaningful endpoint selection (50, 51).

## 7 Conclusions

PICS has emerged as one of the major determinants of long-term morbidity, functional impairment, and reduced quality of life among survivors of sepsis. Despite substantial advances in acute sepsis management, effective therapeutic strategies specifically targeting the persistent immunometabolic disturbances underlying PICS remain lacking. This unmet clinical need highlights the importance of exploring novel therapeutic concepts capable of modulating interconnected inflammatory, immune, and metabolic pathways.

Traditional Chinese medicine has attracted increasing attention as a potential adjunctive approach in critical care because of its multi-component and multi-target characteristics. Clinical studies conducted during the acute phase of sepsis suggest that certain TCM interventions may improve inflammatory responses, immune function, and short-term clinical outcomes, while experimental studies provide mechanistic evidence supporting their potential regulation of NF-κB, AMPK, mitochondrial function, oxidative stress, and other immunometabolic pathways relevant to post-sepsis recovery.

Nevertheless, direct evidence supporting the efficacy of TCM in patients with established PICS remains limited. Most currently available data originate from acute-phase sepsis studies or preclinical investigations, and their applicability to the prolonged recovery phase characteristic of PICS remains uncertain. Furthermore, heterogeneity in diagnostic criteria, study design, patient populations, and outcome measures continues to limit the interpretation and generalizability of existing findings.

Taken together, the available evidence supports biological plausibility but remains insufficient to establish the clinical efficacy of TCM in patients with established PICS. The limited availability of direct evidence underscores the need for, rather than diminishes the value of, a critical synthesis of the existing literature. By integrating fragmented clinical, experimental, and mechanistic evidence, this Mini Review aims to distinguish established findings from biologically plausible hypotheses, clarify the strengths and limitations of the current evidence base, identify key knowledge gaps, and provide a conceptual immunometabolic framework to facilitate future research in this emerging field.

Future investigations should prioritize well-designed multicenter prospective studies incorporating standardized diagnostic criteria for PICS, longitudinal immune phenotyping, metabolomic profiling, pharmacokinetic evaluation, and clinically meaningful long-term functional outcomes. Such studies will be essential to determine whether TCM can move beyond mechanistic plausibility toward evidence-based application in the management of sepsis-associated PICS.
